# Association of *TPH2* polymorphisms with valproic acid efficacy and neurological adverse events in pediatric epilepsy

**DOI:** 10.3389/fphar.2026.1821776

**Published:** 2026-07-13

**Authors:** Jiahao Zhu, Xixuan Wang, Xianhuan Shen, Hanbing Xia, Yaodong He, Qian Liu, Jianping Zhang, Xiaomei Fan, Wenzhou Li

**Affiliations:** 1 Department of Pharmacy, Shenzhen Baoan Women’s and Children’s Hospital, Shenzhen, China; 2 Department of Clinical Pharmacology, College of Pharmacy, Jinan University, Guangzhou, China

**Keywords:** adverse drug reaction, childhood epilepsy, polymorphism, responsiveness, TPH2, valproic acid

## Abstract

**Objective:**

Valproic acid (VPA) response in pediatric epilepsy shows high variability. This study investigated the association of tryptophan hydroxylase-2 (*TPH2*) polymorphisms with VPA efficacy and adverse drug reactions (ADRs) in children.

**Methods:**

In a retrospective cohort of 199 children with epilepsy receiving VPA treatment, *TPH2* rs4570625 and rs1386494 were genotyped using Sequenom MassArray. Efficacy was categorized as uncontrolled seizure *vs.* seizure-free. ADRs were systemically recorded, and steady-state VPA concentrations were measured. Network pharmacology integrated protein-protein interactions, and functional enrichment to elucidate *TPH2*’s role in VPA-related neurotoxicity.

**Results:**

The rs4570625 polymorphism was significantly associated with VPA treatment outcomes. GG homozygotes exhibited higher seizure-free rates (*p* = 0.031) and lower incidence of neurological ADRs (*p* = 0.036). GG carriers also displayed significantly higher VPA serum concentrations. Network pharmacology analyses identified the serotonergic synapse as the dominant enriched pathway linking *TPH2* to VPA neurotoxicity, with *MAOA* and *SLC6A4* as key regulatory hubs. No significant associations were observed between rs1386494 polymorphism and VPA treatment outcomes.

**Conclusion:**

*TPH2* rs4570625 may serve as a potential exploratory biomarker for predicting VPA efficacy and neurological toxicity in pediatric epilepsy, mediated through serotonergic pathway dysregulation. Genotype-guided personalization could optimize VPA therapy.

## Key points

- This study investigated the association between *TPH2* polymorphisms and the efficacy, pharmacokinetics, and ADRs of VPA in pediatric epilepsy.
- Patients with the GG genotype of *TPH2* rs4570625 showed better seizure control, reduced neurotoxicity, and higher serum VPA concentrations compared to carriers of other genotypes.
- The serotonergic synapse may be the key pathway linking *TPH2* function to VPA-induced neurological ADRs, with *MAOA* and *SLC6A4* as key regulatory hubs.

## 1 Introduction

Epilepsy, a chronic brain disorder with multiple etiologies, affects more than 70 million people worldwide (Thijs et al., 2019). Pediatric epilepsy accounts for 20%–40% of all cases, with peak incidence in early childhood, a period of heightened neuronal plasticity (Aaberg et al., 2017). Beyond recurrent seizures, children with epilepsy face elevated risks of cognitive impairment, psychiatric comorbidities, and social stigmatization, compounding the overall disease burden.

Currently, antiseizure medications (ASMs) remain the cornerstone of epilepsy management; however, over 30% of patients develop drug resistance despite optimal drug selection (Kwan and Brodie, 2000). Drug resistance not only leads to uncontrolled seizures, but also increases the risks of physical injury, psychological dysfunction, and reduced quality of life. Additionally, adverse drug reactions (ADRs) are reported in approximately 80% of patients, with an even higher prevalence in pediatric populations, often leading to treatment discontinuation and additional morbidity (Strzelczyk and Schubert-Bast, 2022; Baker et al., 1997).

Valproic acid (VPA), a first-line broad-spectrum ASM, exerts its anticonvulsant effects through multiple mechanisms including GABAergic enhancement, voltage-gated sodium channel inhibition, and histone deacetylase activity modulation (Ghodke-Puranik et al., 2013). Despite its broad efficacy, there is significant variability in individual responses. Some pediatric patients experience suboptimal seizure control or develop dose-dependent ADRs such as hepatotoxicity, tremor and weight gain. This variability is partially explained by pharmacogenetic polymorphisms in drug-metabolizing enzymes (*CYP2C9* and *UGT1A6*) and neuronal targets (*SCN1A*) (Guo et al., 2012; Toth et al., 2015; Wang et al., 2018). Nevertheless, the potential role of serotonin pathway genes like *TPH2* in modulating VPA response has not been systematically investigated, despite the involvement of serotonin in seizure modulation (Bagdy et al., 2007).

Serotonin (5-hydroxytryptamine, 5-HT) modulates neuronal excitability through both presynaptic and postsynaptic receptors (Bagdy et al., 2007). Preclinical evidence demonstrates that 5-HT depletion lowers seizure thresholds, whereas 5-HT agonists exert anticonvulsant effects (Statnick et al., 1996a). Tryptophan hydroxylase-2 (TPH2), the rate-limiting enzyme in central 5-HT synthesis, plays a crucial role in maintaining physiological serotonergic neurotransmission (Walther et al., 2003). Genetically epilepsy-prone rats exhibit decreased 5-HT levels and reduced tryptophan hydroxylase activity (Statnick et al., 1996b), supporting a link between TPH2 function and seizure susceptibility. In humans, *TPH2* polymorphisms (particularly rs4570625 and rs1386494) have been associated with various neuropsychiatric disorders including major depression (MD), tic disorder (TD), and attention-deficit/hyperactivity disorder (ADHD) (Zill et al., 2004; Zheng et al., 2013; Walitza et al., 2005). The rs4570625 variant has been associated with psychiatric comorbidities in patients with epilepsy (Bragatti et al., 2014). Notably, a recent positron emission tomography study demonstrated that rs1386494 significantly affects cerebral serotonin turnover, with CC homozygotes showing 26% higher MAO-A levels (Spies et al., 2023), confirming the functional relevance of these variants in the human serotonergic system. Although VPA is not a direct TPH2 target, its pharmacological effects include modulation of serotoninergic transmission (Perucca, 2002), and animal studies have shown that VPA administration alters brain 5-HT metabolism (Hwang and Van Woert). Pharmacologically, selective serotonin reuptake inhibitors can elevate brain concentrations of VPA (Borowicz et al., 2007). Given that TPH2 controls 5-HT synthesis, genetic variation in *TPH2* may influence serotonergic level, thereby potentially modulating VPA’s efficacy and ADR profile. However, direct evidence linking *TPH2* polymorphisms to VPA response remains limited, warranting the present investigation.

Pharmacogenomics identifies genetic variants underlying interindividual differences in drug efficacy and toxicity, whereas, network pharmacology integrates multi-omics data to elucidate gene-pathway-disease interactions within biological systems. By combining these complementary approaches with our previous research (Fan et al., 2020; Zhu et al., 2023a; Fan et al., 2021; Zhu et al., 2023b; Shen et al., 2025), we investigated the effects of *TPH2* polymorphisms (rs4570625 and rs1386494) on VPA efficacy, pharmacokinetics, and ADRs in pediatric epilepsy. The selection of *TPH2* polymorphisms was guided by previous studies suggesting their involvement in neuropsychiatric disorders and drug responses (Zill et al., 2004; Zheng et al., 2013; Walitza et al., 2005; Bragatti et al., 2014). Subsequent network analyses systematically elucidated the *TPH2*-centric pathways and core functional networks involved in VPA-induced ADRs, providing mechanistic insights that could facilitate precision medicine for pediatric epilepsy.

## 2 Materials and methods

### 2.1 Subjects

A total of 208 Chinese pediatric epilepsy patients were included in this study from the Department of Paediatrics, Shenzhen Baoan Women’s and Children’s Hospital between January 2017 and December 2023. The inclusion criteria were as follows: (1) diagnosis of epilepsy or an epileptic syndrome confirmed by clinical and electroencephalographic (EEG) criteria (Scheffer et al., 2017); (2) age at diagnosis ranging from 1 month to 16 years; and (3) continuous VPA treatment for at least 1 year. The exclusion criteria were as follows: (1) significant hepatic or renal impairment; (2) concomitant medications known to significantly affect VPA metabolism; (3) diagnosis of epilepsy within the first month of life; (4) Dravet syndrome or significant structural brain abnormalities such as malformations of cortical development, tumors, or traumatic brain injury; or (5) insufficient clinical or diagnostic documentation.

We retrospectively collected demographic and clinical data including sex, age, body weight, seizure type, medication regimen, VPA serum concentration, seizure control status, and occurrence of ADRs through the hospital information system or structured telephone interviews. Specifically, to capture ADR information, we reviewed medical records and conducted structured telephone interviews with caregivers at multiple time points during the 12-month treatment period, rather than relying on a single post-treatment assessment. Where available, caregiver-maintained diaries completed during the treatment period were also reviewed. All telephone interviews followed a standardized questionnaire that prompted caregivers to recall ADRs by specific time intervals during VPA therapy to aid recall accuracy. The study protocol was approved by the Ethics Committee of Shenzhen Baoan Women’s and Children’s Hospital (protocol code: LLSC 2020-10–06-KS). Written informed consent was obtained from the parents or legal guardians of all participants prior to enrolment.

### 2.2 Serum VPA sampling and quantification

All VPA serum concentration measurements were obtained from routine therapeutic drug monitoring records within the hospital information system. For each measurement, we retrospectively confirmed from medical records that patients had maintained a stable VPA dosage for at least 5 days prior to blood sampling. All samples were collected in the morning before the first daily dose following an overnight fast of at least 8 h. Samples were centrifuged at 3000 rpm for 10 min to obtain serum. Free VPA serum concentrations were quantified using homogeneous enzyme immunoassay (HEIA) on a Hitachi biochemical analyzer (7180 HITACHI, Tokyo, Japan). The intra-assay and inter-assay coefficients of variation for the HEIA method were maintained below 8.0% and 10.0%, respectively. To normalize exposure for body weight and daily dose, the concentration-to-dose ratio (CDR) was calculated as: CDR (µg·mL^-1^·mg^-1^·kg) = serum VPA concentration (µg/mL)/[daily VPA dose (mg)/weight (kg)] (Wang et al., 2016).

### 2.3 Assessment of VPA efficacy and ADRs

Based on predefined criteria (Kwan et al., 2010), patients were categorized into two groups: the uncontrolled seizure group and the seizure-free group. The uncontrolled seizure group comprised patients who failed to achieve seizure freedom despite receiving VPA monotherapy or combination therapy with other ASMs for at least 12 months at the maximum tolerated dose. The seizure-free was defined as the absence of any type of seizure for a minimum of 1 year during VPA treatment (Fan et al., 2020; Zhu et al., 2023a). Of note, in the context of this study, “uncontrolled” refers to any ongoing seizure occurrence during the 12-month follow-up period, rather than strictly adhering to the formal definition of drug-resistant epilepsy. ADRs associated with VPA were systematically evaluated through a combination of medical record reviews, telephone interviews, and caregiver-maintained diaries. Patients were stratified by ADR occurrence: those with ADRs and those without ADRs. The ADRs group was subclassified into four categories according to the involved systems: neurological ADRs (nADRs), gastrointestinal ADRs (gADRs), cutaneous ADRs (cADRs), and weight gain (Johannessen and Johannessen, 2003). The causality of ADRs was evaluated using the standardized and widely recognized WHO-UMC causality assessment scale (Fan et al., 2021; Shukla et al., 2021), with only certain, probable/likely, or possible cases retained for analysis to ensure data reliability.

### 2.4 Genotyping

Two single nucleotide polymorphisms (SNPs) in the *TPH2* gene including rs4570625 and rs1386494 were selected from the National Center for Biotechnology Information (NCBI) dbSNP database (https://www.ncbi.nlm.nih.gov/snp/). These SNPs were chosen based on documented associations with neuropsychiatric disorders and pharmacotherapeutic response variability (Zill et al., 2004; Zheng et al., 2013; Walitza et al., 2005; Bragatti et al., 2014). Peripheral venous blood samples (1–1.5 mL) were collected from each participant using EDTA-coated tubes to prevent coagulation. Genomic DNA was extracted from whole blood using a standardized protocol as previously described (Loparev et al., 1991). DNA samples were amplified using polymerase chain reaction (PCR) under standardized conditions as described in our previous studies (Fan et al., 2020; Zhu et al., 2023a). Briefly, the 5 µL PCR reaction system contained 10 ng of DNA template and 0.5 µM of each primer. The thermal cycling conditions were as follows: initial denaturation at 95 °C for 2 min, followed by 45 cycles of denaturation at 95 °C for 30 s, annealing at 56 °C for 30 s, and extension at 72 °C for 60 s, with a final extension at 72 °C for 5 min. The amplified products were then purified using shrimp alkaline phosphatase according to the manufacturer’s instructions. Genotyping of the *TPH2* SNPs (rs4570625 and rs1386494) was performed using the Sequenom MassArray system (Agena Bioscience, San Diego, CA, United States) with the iPLEX Gold Assay. Genotyping results were analyzed using MassArray Analyzer mass spectrometer and integrated data analysis software. The specific primers for the studied SNPs were designed for this study as follows: forward primer 5′-ACGTTGGATGACTCACACATTTGCATGCAC-3′and reverse primer 5′-ACG​TTG​GAT​GGA​TCT​TAT​CCC​TCC​CAT​CAG-3′ for rs4570625; forward primer 5′-ACG​TTG​GAT​GAA​TCA​ATT​GCC​AGG​GAT​GTG-3′ and reverse primer 5′- ACG​TTG​GAT​GCA​AAT​GAA​TCA​CAA​AGG​GTG​C-3′ for rs1386494. Genotyping call rate exceeded 95% for both SNPs, and 10% of samples were randomly selected for duplicate genotyping with 100% concordance.

### 2.5 Protein-protein interaction analysis

To explore the functional network of *TPH2* in VPA-induced ADRs, protein-protein interaction (PPI) analysis was performed using the STRING database (https://string-db.org/) (Szklarczyk et al., 2021). The *TPH2* gene was input into STRING with the species set to *Homo sapiens*, an interaction confidence score ≥0.4, and a maximum of 50 interacting genes. The resulting PPI network was visualized and analyzed using Cytoscape software (v3.9.1).

### 2.6 Identification of *TPH2*-related target genes in VPA-induced ADRs

VPA-associated genes were retrieved from DrugBank (https://go.drugbank.com/) and GeneCards (https://www.genecards.org/) database (Wishart et al., 2018; Safran et al., 2021). Genes with a GeneCards relevance score ≥1 were selected. Duplicates were removed to generate a comprehensive list of VPA-associated genes. Additionally, genes linked to VPA-induced ADRs (e.g., asthenia, somnolence, sleep disorders, headache and irritability) were extracted from GeneCards and DisGeNET database (https://www.disgenet.org/) (Pinero et al., 2021). The filtering criteria were set as the number of PubMed IDs (PMIDs) > 0 for DisGeNET and the relevance score ≥1 for GeneCards. To identify potential *TPH2*-related target genes involved in VPA-induced ADRs, the overlapping genes among VPA-associated genes, ADR-related genes, and genes from *TPH2* PPI network were determined.

### 2.7 Pathway and functional enrichment analysis

The Metascape platform (https://metascape.org/gp/) was employed to perform Gene Ontology (GO) and Kyoto Encyclopedia of Genes and Genomes (KEGG) pathway enrichment analysis (Zhou et al., 2019). The parameters were set as follows: species = *H. sapiens*, minimum enrichment count = 3, p-value ≤0.01, and enrichment factor >1.5. Enriched terms were ranked by significance to elucidate the *TPH2*-associated pathways and biological processes potentially involved in VPA-induced ADRs. The results were visualized using a bioinformatics online tool (http://www.bioinformatics.com.cn).

### 2.8 Statistical analysis

Statistical analysis was performed using IBM SPSS Statistics (version 26). The normality of continuous variables was assessed using the Kolmogorov-Smirnov test. Normally distributed data were expressed as mean ± standard deviation (SD) and compared between groups using independent samples *t*-test. Non-normally distributed data were presented as median (1st-3rd quartiles) and analyzed with Mann-Whitney *U* test. Categorical variables were summarized as frequencies (%) and compared b*et*ween groups using chi-square (χ^2^) tests or Fisher’s exact test as appropriate. For genetic analyses, allele and genotype frequencies were compared using the SNPStats online tool (https://www.snpstats.net/), with wild-type as reference. Deviations from Hardy-Weinberg equilibrium (HWE) were assessed using χ^2^ tests. Post-hoc power analysis was performed using G*Power (version 3.1.9.7) with the goodness-of-fit test for contingency tables. Differences in steady-state VPA serum concentrations across genotypes were evaluated using one-way ANOVA with Bonferroni’s *post hoc* tests or Kruskal–Wallis *H* test. All tests were two-tailed, and a *p*-value <0.05 was considered statistically significant. As a sensitivity analysis, Bonferroni correction was applied for the three primary hypothesis-driven comparisons (efficacy, neurological ADRs, and CDR). The corrected significance threshold was α = 0.0167 (0.05/3). Findings with p < 0.05 but above this threshold were considered exploratory.

## 3 Results

### 3.1 Clinical characteristics of children with epilepsy

We included 208 pediatric patients with epilepsy who were treated with VPA. After excluding 9 patients with Dravet syndrome or structural brain lesions, 199 patients were retained for the final analysis (Table 1). Of these, 122 patients achieved seizure freedom, whereas 77 continued to have uncontrolled seizures. The median age at diagnosis was 3.42 years, and 60.8% of the cohort were male. Generalized-onset seizures predominated, followed by focal-onset and unknown-onset seizures. No significant differences in age, gender, or seizure type were observed between the uncontrolled seizure and seizure-free groups. Notably, polytherapy was more frequently administered in the uncontrolled seizure group, whereas VPA monotherapy predominated in seizure-free patients. Levetiracetam (27.6%) and oxcarbazepine (20.6%) were the most frequent concomitant ASMs. A total of 210 ADRs related to VPA were recorded. Gastrointestinal ADRs and neurological ADRs were the most frequent, followed by weight gain and cutaneous ADRs.

**TABLE 1:** Clinical characteristics of children with epilepsy treated with VPA.

Characteristics	Total (n = 199)	Uncontrolled Seizure (n = 77)	Seizure-Free (n = 122)	*P*-value^d^
Age at diagnosis				
1 month–2 years	78 (39.2%)	30 (39.0%)	48 (39.3%)	0.957
2–16 years	121 (60.8%)	47 (61.0%)	74 (60.7%)	​
Median (quartile 1–3)	3.42 (1.08–6.08)	3.33 (1.34–5.21)	3.50 (1.00–6.92)	0.322
Gender				
Male	121 (60.8%)	52 (67.5%)	69 (56.6%)	0.122
Female	78 (39.2%)	25 (32.5%)	53 (43.4%)	​
Seizure type				
Generalized onset	125 (62.8%)	48 (62.3%)	77 (63.1%)	0.951
Focal onset	60 (30.2%)	24 (31.2%)	36 (29.5%)	​
Unknown onset	14 (7.0%)	5 (6.5%)	9 (7.4%)	​
Subtype of epilepsy^a^				
Generalized tonic-clonic seizure	76 (38.2%)	31 (40.3%)	45 (36.9%)	0.633
Tonic seizure	9 (4.5%)	3 (3.9%)	6 (4.9%)	1.000
Clonic seizure	10 (5.0%)	1 (1.3%)	9 (7.4%)	0.114
Myoclonic seizure	4 (2.0%)	3 (3.9%)	1 (0.8%)	0.323
Childhood absence epilepsy	17 (8.5%)	5 (6.5%)	12 (9.8%)	0.411
Focal seizures	39 (19.6%)	15 (19.5%)	24 (19.7%)	0.974
Benign epilepsy in childhood with centrotemporal spikes	16 (8.0%)	6 (7.8%)	10 (8.2%)	0.919
Others	28 (14.1%)	13 (16.9%)	15 (12.3%)	0.365
ASM therapy				
VPA monotherapy	106 (53.3%)	21 (27.3%)	85 (69.7%)	<0.01^**^
Polytherapy	93 (46.7%)	56 (72.7%)	37 (30.3%)	​
Levetiracetam	55 (27.6%)^b^	36 (46.8%)	19 (15.6%)	​
Oxcarbazepine	41 (20.6%)	23 (29.9%)	18 (14.8%)	​
Others	28 (14.1%)	18 (23.4%)	10 (8.2%)	​
Adverse drug reactions				
With ADRs	132 (66.3%)	51 (66.2%)	81 (66.4%)	0.981
Without ADRs	67 (33.7%)	26 (33.8%)	41 (33.6%)	​
Neurological ADRs	61 (29.0%)^c^	​	​	​
Gastrointestinal ADRs	69 (32.9%)	​	​	​
Weight gain	58 (27.6%)	​	​	​
Cutaneous ADRs	22 (10.5%)	​	​	​

Abbreviations: ADRs, adverse drug reactions; ASMs, antiseizure medications; VPA, valproic acid.

^a^ For patients with multiple seizure subtypes, the predominant subtype was used for classification.

^b^ Due to the concomitant use of multiple ASMs, the total number of patients across different ASM, categories exceeds the size of the overall sample.

^c^ The total number of patients across various ADR, categories exceeds the total sample size because some patients experienced more than one ADR.

^d^ The *p*-value was calculated by comparing the uncontrolled seizure group (n = 77) with the seizure-free group (n = 122). **p < 0.01

The distributions of *TPH2* polymorphisms (rs4570625 and rs1386494) across patient clinical characteristics were summarized in Supplementary Table S1. For rs4570625, genotype frequencies were GG 22.1%, GT 49.8%, and TT 28.1%. For rs1386494, the frequencies were TC 11.1% and CC 88.9%. In terms of genotype distribution, there was a significant sex difference for rs4570625 (*p* = 0.001), while rs1386494 showed a significant variation with age at diagnosis (*p* = 0.043). Neither polymorphism was associated with seizure types or ASM therapy regimens.

### 3.2 The association of *TPH2* polymorphisms with VPA responsiveness in children with epilepsy

Post-hoc power analysis revealed that for rs4570625 (effect size w = 0.22, α = 0.05), the achieved power was 0.99. For rs1386494 (w = 0.04), the achieved power was 0.15, indicating insufficient statistical power to detect a small effect for this variant. The *TPH2* rs4570625 polymorphism demonstrated significant associations with VPA response in pediatric patients (Table 2). The genotype distributions for both rs4570625 and rs1386494 conformed to HWE (*p* > 0.05). Allele frequency analysis revealed a significantly higher T allele frequency in the uncontrolled seizure group (*p* = 0.033). Genotype analysis showed that GG homozygotes were more frequent in the seizure-free group, whereas TT carriers predominated in the uncontrolled seizure group. Under the dominant genetic model (GG *vs.* GT + TT), carriers of GG genotype exhibited better treatment response (*p* = 0.031). In contrast, rs1386494 showed no significant association with VPA efficacy (Supplementary Table S2). Because linkage disequilibrium between the two SNPs was weak, haplotype analysis was not performed.

**TABLE 2:** Distribution of *TPH2* rs4570625 genotypes in uncontrolled seizure and seizure-free groups.

Genetic Model	Genotype	Uncontrolled seizure (n = 77)	Seizure-free (n = 122)	OR (95%CI)	*p*-value
Allele contrast	G *vs.* T	62 (40.3%)	125 (51.2%)	1.00	0.033*
​	​	92 (59.7%)	119 (48.8%)	0.64 (0.43–0.97)	​
Codominant	GG *vs.* GT *vs.* TT	11 (14.3%)	33 (27.0%)	1.00	0.074
​	​	40 (51.9%)	59 (48.4%)	0.49 (0.22–1.09)	​
​	​	26 (33.8%)	30 (24.6%)	0.38 (0.16–0.91)	​
Dominant	GG *vs.* GT + TT	11 (14.3%)	33 (27.0%)	1.00	0.031*
​	​	66 (85.7%)	89 (73.0%)	0.45 (0.21–0.95)	​
Recessive	GG + GT *vs.* TT	51 (66.2%)	92 (75.4%)	1.00	0.160
​	​	26 (33.8%)	30 (24.6%)	0.64 (0.34–1.20)	​
Overdominant	GG + TT *vs.* GT	37 (48.1%)	63 (51.6%)	1.00	0.620
​	​	40 (51.9%)	59 (48.4%)	0.87 (0.49–1.53)	​

* *P* < 0.05.

### 3.3 The association of *TPH2* polymorphisms with VPA-Induced ADRs in children with epilepsy

We further explored association whether *TPH2* variants influence the occurrence of VPA-related ADRs. A significant association was observed between *TPH2* rs4570625 and VPA-induced nADRs (Table 3). There were 61 patients who experienced nADRs related to VPA. Allele frequency analysis demonstrated no significant differences in the distribution of rs4570625 between patients with and without nADRs. However, under the dominant genetic model, the GG genotype showed significantly higher prevalence in patients without nADRs (*p* = 0.036), suggesting a potential protective effect against VPA-induced nADRs. Neither rs4570625 nor rs1386494 was associated with other VPA-related ADRs (Supplementary Tables S3-S7).

**TABLE 3:** Distribution of *TPH2* rs4570625 genotypes in the groups of patients with and without nADRs.

Genetic Model	Genotype	Patients with nADRs (n = 61)	Patients without nADRs (n = 147)	OR (95%CI)	*p*-value
Allele contrast	G *vs*. T	51 (41.8%)	144 (49.0%)	1.00	0.180
​	​	71 (58.2%)	150 (51.0%)	0.75 (0.49–1.15)	​
Codominant	GG *vs*. GT *vs*. TT	8 (13.1%)	38 (25.9%)	1.00	0.100
​	​	35 (57.4%)	68 (46.3%)	0.41 (0.17–0.97)	​
​	​	18 (29.5%)	41 (27.9%)	0.48 (0.19–1.23)	​
Dominant	GG *vs*. GT + TT	8 (13.1%)	38 (25.9%)	1.00	0.036*
​	​	53 (86.9%)	109 (74.1%)	0.43 (0.19–0.99)	​
Recessive	GG + GT *vs*. TT	43 (70.5%)	106 (72.1%)	1.00	0.810
​	​	18 (29.5%)	41 (27.9%)	0.92 (0.48–1.78)	​
Overdominant	GG + TT *vs*. GT	26 (42.6%)	79 (53.7%)	1.00	0.140
​	​	35 (57.4%)	68 (46.3%)	0.64 (0.35–1.17)	​

Abbreviations: nADRs, neurological adverse drug reactions.

* P < 0.05.

### 3.4 The association of *TPH2* polymorphisms with serum VPA concentrations

A total of 500 steady-state VPA concentration were collected from 175 patients (Table 4). The uncontrolled seizure group required a significantly higher daily dose than the seizure-free group (*p* = 0.029). When classified into concentration ranges (<50, 50–100, >100 μg/mL), no significant distributional differences were observed between groups. However, after adjustment for body weight and dose using the CDR, seizure-free patients exhibited significantly higher CDR values (*p* = 0.002), suggesting more efficient drug exposure.

**TABLE 4:** Distribution of VPA serum concentrations in uncontrolled seizure and seizure-free groups.

Characteristics	Uncontrolled seizure	Seizure-free	*p*-value
Number of patients	66	109	​
Number of VPA concentration samples	193	307	​
Daily dose (mg/kg)	23.5 ± 7.4	22.2 ± 6.8	0.029*
VPA serum concentration (μg/mL)	​	​	​
<50	55 (28.5%)	83 (27.0%)	0.602
50–100	130 (67.4%)	205 (66.8%)	0.832
>100	8 (4.1%)	19 (6.2%)	0.317
Mean ± SD	60.5 ± 22.2	64.3 ± 21.7	0.063
VPA CDR (μg·kg/mL·mg)	2.5 (2.0–3.1)^a^	2.8 (2.1–3.7)	0.002**

Abbreviations: SD, standard deviation; CDR, Concentration-to-dose ratio; VPA, valproic acid.

* *P* < 0.05,

** *P* < 0.01.

^a^ Median (quartile 1–3).

We further investigated the influence of *TPH2* polymorphisms on VPA pharmacokinetics. Genotype analysis revealed a significant effect of rs4570625 on serum VPA levels and GG homozygotes displayed higher concentrations than GT or TT carriers (*p* = 0.043) (Table 5). This difference became even more pronounced when CDR was used as the outcome. GG individuals had significantly elevated CDR values (*p* < 0.01), suggesting enhanced drug exposure efficiency. In contrast, rs1386494 showed only a borderline association with serum VPA concentrations and no significant effect on CDR. After adjusting for three primary hypothesis-driven comparisons (α = 0.0167), the association between the rs4570625 GG genotype and higher CDR remained statistically significant (*p* < 0.01). However, the associations with VPA efficacy (*p* = 0.031) and neurological ADR protection (*p* = 0.036) did not reach the adjusted significance threshold. These results suggest that the pharmacokinetic parameter CDR is a more robust indicator of genotype-dependent VPA exposure, while the efficacy and safety findings should be interpreted as preliminary and hypothesis-generating.

**TABLE 5:** Comparison of VPA serum concentration and CDR by *TPH2* genotypes in epilepsy patients.

Gene	SNP	Genotype	VPA Concentration (μg/mL)	VPA CDR (μg·kg/mL·mg)
*TPH2*	rs4570625	GG (23.2%)^a^	67.1 ± 19.8^b^	3.0 (2.4–3.7)^c^
		GT (46.4%)	60.8 ± 22.5	2.5 (1.9–3.2)
		TT (30.4%)	62.7 ± 22.3	2.7 (2.0–3.5)
		*p*-value	0.043* ^d^	< 0.01** ^e^
	rs1386494	TC (10.4%)	56.5 ± 20.9	2.6 (1.9–3.4)
		CC (89.6%)	63.6 ± 22.0	2.6 (2.1–3.4)
		*p*-value	0.028* ^f^	0.437^g^

Abbreviations: CDR, Concentration-to-dose ratio; SNP, single nucleotide polymorphism; VPA, valproic acid.

* P < 0.05,

** P < 0.01.

^a^ Percentages in this table are based on the number of VPA, concentration measurements (n = 500), not the number of patients.

^b^ Mean ± SD.

^c^ Median (quartile 1–3).

^d^ One-way ANOVA.

^e^ Kruskal–Wallis H test.

^f^ Independent samples t-test.

^g^ Mann-Whitney U test.

### 3.5 Network pharmacology analysis of *TPH2* in VPA-induced nADRs

**FIGURE 1:**
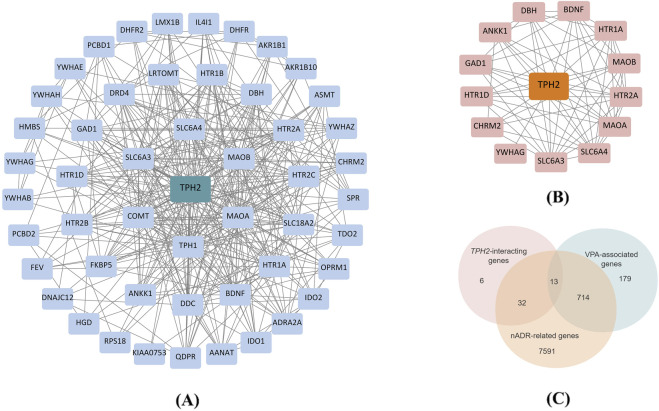
Protein-protein interaction (PPI) network analysis. **(A)** The PPI network of *TPH2* consisted of 51 nodes and 372 edges. **(B)** The key subnetwork of 13 core genes associated with *TPH2* in VPA-induced nADRs. Each target protein is represented as a node, and the association between two proteins is denoted by lines. **(C)** The Venn graph of *TPH2*-interacting genes, VPA-associated genes, and nADR-related genes.

To elucidate the mechanistic role of *TPH2* in VPA-induced nADRs, we integrated PPI and multi-database screening. Using the STRING database and Cytoscape, we extracted 50 *TPH2*-interacting genes, constructing a PPI network of 51 nodes and 372 edges (Figure 1A). From DrugBank and GeneCards databases, 906 VPA-associated genes were identified. Besides, 8350 nADR-related genes were obtained from GeneCards and DisGeNET after deduplication. The intersection of these datasets identified 13 key targets linking *TPH2* with VPA-induced nADRs, including *MAOA*, *MAOB*,*SLC6A4*,*BDNF*,*SLC6A3*, *HTR1A*,*HTR2A*,*DBH*,*GAD1*, *HTR1D*, *ANKK1*,*CHRM2*,*YWHAG* (Figures 1B,C).

**FIGURE 2:**
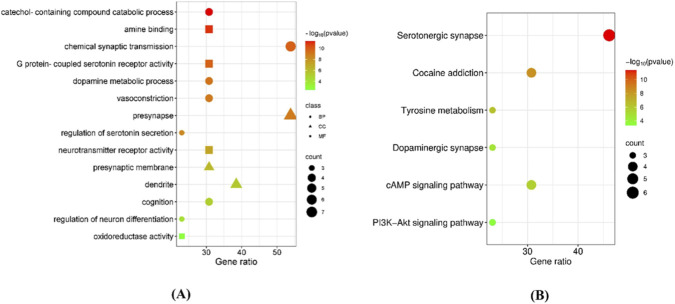
Gene Ontology (GO) enrichment and Kyoto Encyclopedia of Genes and Genomes (KEGG) pathway analysis of 13 common genes. **(A)** GO enrichment analysis of 13 potential target genes of *TPH2* in VPA-induced nADRs. Polka dots, triangles, and squares represent biological processes (BP), cellular components (CC), and molecular functions (MF), respectively. **(B)** KEGG pathway analysis. Gene ratio refers to the ratio of enriched genes to all target genes, and the counts refer to the number of the enriched genes.

GO and KEGG analysis via Metascape uncovered biological function and pathway of the 13 candidate genes. Top enriched biological processes (BP) included catechol-containing compound catabolic process, chemical synaptic transmission, and regulation of serotonin secretion. Cellular components (CC) highlighted presynaptic membrane and dendrite, while molecular functions (MF) involved amine binding, G protein-coupled serotonin receptor activity, and neurotransmitter receptor activity (Figure 2A). KEGG pathway analysis further identified neuroactive pathways: serotonergic synapse, cocaine addiction, tyrosine metabolism, and dopaminergic synapse as most significant (Figure 2B). Collectively, these results implicated *TPH2* in modulating VPA-induced nADRs through serotonergic and dopaminergic neurotransmission pathways.

## 4 Discussion

This study investigated the role of *TPH2* polymorphisms in VPA response and ADRs in children with epilepsy. We found that the rs4570625 variant is significantly associated with VPA treatment outcomes. Children with the GG genotype achieved higher seizure-free rates, displayed lower neurotoxicity, and maintained both higher serum VPA concentrations and CDR compared to carriers of other genotypes. Network-pharmacology analyses further identified the serotonergic synapse as the key pathway linking *TPH2* function to VPA-induced neurotoxicity. Clinically, 38.7% of our cohort had inadequate seizure control and 66.3% experienced ADRs, primarily neurological and gastrointestinal events, which were consistent with previous reports (Li et al., 2016; Nanau and Neuman, 2013).

The significant association between *TPH2* rs4570625 variant and VPA efficacy/pharmacokinetics likely reflects 5-HT-mediated neuromodulation. Epileptic seizures emerge when the balance between excitation and inhibition is disrupted, and 5-HT serves as a key inhibitory neurotransmitter (Akyuz et al., 2021). Preclinical evidence demonstrates that anticonvulsant treatment with phenytoin elevates cerebral 5-HT levels in rat epilepsy models (Bonnycastle et al., 1957), and subsequent work confirm that increased 5-HT signaling suppresses seizures, whereas 5-HT depletion lowers seizure threshold (Statnick et al., 1996a; Pasini et al., 1996). As the rate-limiting enzyme in central 5-HT synthesis, *TPH2* directly regulates epileptogenic processes. Genetically epilepsy-prone rats display widespread serotonergic terminal loss, reduced 5-HT levels, and diminished tryptophan hydroxylase (TPH) activity (Statnick et al., 1996b), supporting the hypothesis that altered TPH2 function contributes to seizure susceptibility.

Chronic treatment with serotonin reuptake inhibitors significantly elevates brain concentrations of VPA and phenytoin, consequently enhancing antiepileptic efficacy (Borowicz et al., 2007). Microdialysis studies in rats have demonstrated that VPA increases extracellular serotonin in the hippocampus and striatum (Biggs et al., 1992), and VPA-treated epileptic mice exhibit significantly elevated 5-HT metabolites and heightened TPH activity in the striatum and midbrain tegmentum (Vriend and Alexiuk, 1996). In human studies, the rs4570625 T allele has been linked to psychiatric comorbidities in people with epilepsy (Bragatti et al., 2014). Consistent with these observations, our cohort showed that GG homozygotes of *TPH2* rs4570625 achieved better seizure control and higher VPA exposure, suggesting that GG genotype may affect 5-HT synthesis and enhance the antiepileptic effects of VPA. The rs4570625 (−703 G>T) polymorphism is located within the promoter region of the *TPH2* gene (Chen and Miller, 2012). Functionally, the T allele disrupts transcription factor binding, lowering TPH2 expression and reducing cerebral 5-HT synthesis (Chen et al., 2008). This deficiency triggers compensatory prefrontal hyperactivity during emotional processing, which may explain reduced seizure control and heightened nADRs susceptibility in T carriers. This genetic modulation of enzyme activity could explain the pharmacokinetic differences, as optimal 5-HT levels may potentiate VPA’s neuronal targets. Future studies should directly quantify brain 5-HT dynamics relative to *TPH2* genotypes to validate this model.

Beyond efficacy, *TPH2* rs4570625 significantly modulates VPA-induced nADRs, expanding its established role in neuropsychiatric disorders. Given the critical role of TPH2 in cerebral 5-HT synthesis, this rate-limiting enzyme has been implicated in various neuropsychiatric disorders, including MD, anxiety disorders, schizophrenia, mood disorders, and bipolar disorder (Ottenhof et al., 2018). Tryptophan depletion leads to a transient decrease in cerebral 5-HT levels and may trigger functional variations in the upstream regulatory regions of *TPH2* (SNP rs4570625) (Canli et al., 2005). Functional magnetic resonance imaging evidence indicates that the T allele of this common regulatory variant enhances amygdala reactivity, a core structure governing the regulation of emotional behaviors (Brown et al., 2005). Additional evidence confirms that *TPH2* rs4570625 possesses important biological functions. found that carriers of the rs4570625 T allele obtained higher positive symptom scores for schizophrenia Zhang et al. (2011). Additionally, infants with the T-carrier genotype have been reported to exhibit more pronounced attention-shifting deficits (Leppänen et al., 2011). Our findings are consistent with previous conclusions: GG homozygotes showed a lower risk of VPA-induced nADRs. Network pharmacology elucidated the polygenic landscape underlying VPA-induced nADRs, revealing how *TPH2* interfaces with complementary pathways through systematic bioinformatic integration. Mechanistically, network pharmacology identified serotonergic synapse disruption as the central pathway, with *MAOA*, *MAOB*, and *SLC6A4* emerging as pivotal hubs. The *MAOA* and *MAOB* genes encode enzymes that catalyze the degradation of 5-HT into inactive metabolites, while *SLC6A4* mediates signal termination via presynaptic reuptake mechanisms (Correia and Vale, 2022). These genes collectively regulate 5-HT metabolism, synergizing with *TPH2*-driven synthesis deficits to amplify neurotoxicity. Notably, the dopaminergic synapse and cocaine addiction pathways further implicated reward circuitry dysregulation in VPA-induced irritability and sleep disturbances. Network pharmacology analyses provided mechanistic insights that explain our genetic findings at a systems level. This systems-level model transcends single-gene analysis by demonstrating how *TPH2* variants interfere with integrated neurotransmitter networks, mechanistically linking genetic susceptibility to clinical neurotoxicity.

Our findings indicate that *TPH2* rs4570625 as a promising biomarker for predicting VPA treatment response in pediatric epilepsy. Children with the GG genotype exhibit both superior antiepileptic efficacy and a lower risk of nADRs, potentially enabling clinicians to identify optimal VPA candidates and to flag high-risk patients who should receive alternative ASMs. Mechanistically, our study suggests that elevated 5-HT levels appear to potentiate the antiepileptic activity of VPA while mitigating neurotoxicity. This provides novel insights into the treatment strategy of co-administering serotonin reuptake inhibitors to T allele carriers of rs4570625 to reduce the risk of VPA-induced nADRs. Nevertheless, after applying Bonferroni correction for multiple comparisons, only the association with CDR remained statistically significant (*p* < 0.01). The *p*-values for efficacy and nADR protection fell below 0.05 but above the adjusted threshold (0.0167), suggesting that these effects are modest and may be influenced by sample size limitations. Therefore, these findings should be considered hypothesis-generating and require validation in larger, independent cohorts. Several limitations of this study should be acknowledged. First, the retrospective design inherently limits causal inference, and the identified associations should be interpreted as correlational rather than causal. Caregivers may have underreported or overreported ADRs, particularly for less severe or transient events. Second, potential confounding factors such as concomitant medications, dosage adjustments, and treatment adherence were not systematically controlled, although they were documented through medical records and caregiver interviews. Unmeasured confounding may have influenced the observed associations, particularly given the heterogeneity of treatment regimens in real-world clinical practice. Additionally, the heterogeneity of epilepsy etiologies was not systematically considered. Patients with specific genetic epilepsies may exhibit differential responses to VPA, potentially confounding the observed associations. Critically, the absence of cerebrospinal fluid 5-HT measurements prevents direct validation of the proposed transcriptional mechanism of rs4570625. The modest sample size limited statistical power for variants with low minor allele frequency. For rs1386494, *post hoc* power analysis revealed only 15% power to detect the observed small effect (w = 0.04), suggesting that the null finding may reflect insufficient power rather than biological irrelevance. Conversely, for rs4570625, the study achieved adequate power (99%) to detect the observed moderate effect (w = 0.22), supporting the robustness of this positive association.

In summary, this study indicates that *TPH2* rs4570625 is associated with the therapeutic response to VPA in children with epilepsy. The GG genotype not only predicts better seizure control but also confers protection against neurological adverse effects, likely through enhanced serotonergic neurotransmission. Based on these findings, future research should focus on prospective validation in larger, ethnically diverse cohorts to confirm its predictive value. Mechanistic studies using animal models could elucidate how *TPH2*-mediated 5-HT synthesis regulates the neuroactive properties of VPA. Additionally, expanding the study to other key genes in the serotonin pathway may uncover additional pharmacogenetic factors, thereby optimizing VPA therapy. Together, these efforts will advance personalized treatment strategies that maximize therapeutic efficacy while minimizing neurotoxicity risks in vulnerable pediatric populations.

## 5 Conclusion

This study demonstrated that *TPH2* rs4570625 was significantly associated with VPA response in pediatric epilepsy. The GG genotype of rs4570625 correlated with improved seizure control, reduced neurological ADRs, and higher VPA exposure levels, possibly through enhanced serotonergic neurotransmission. Our integrated pharmacogenomic and network pharmacology approach revealed the critical role of serotonergic synapse pathway in VPA-induced nADRs. These findings suggest that *TPH2* rs4570625 may serve as a potential exploratory biomarker for predicting VPA efficacy and neurological toxicity in pediatric epilepsy. However, given the exploratory nature of the study and the marginal significance of some findings after multiple testing correction, these results require validation in prospective, larger cohorts before clinical translation.

## Data Availability

The raw data supporting the conclusions of this article will be made available by the authors, without undue reservation.
