# Supplementary material for: Association of TPH2 polymorphisms with valproic acid efficacy and neurological adverse events in pediatric epilepsy
Source: Front Pharmacol. 2026 Jul 13;17:1821776. doi: 10.3389/fphar.2026.1821776 (PMC13402418; doi:10.3389/fphar.2026.1821776)
Supplement: Supplementary file 1 [file Table1.docx]

**Table S1** Associations between *TPH2* polymorphisms and clinical characteristics of the patients

| **Characteristics** | ***TPH2*** **rs4570625** | | | ***p*-value** | ***TPH2*** **rs1386494** | | ***p*-value** |
| --- | --- | --- | --- | --- | --- | --- | --- |
|  | **GG** | **GT** | **TT** |  | **TC** | **CC** |  |
| **Age at diagnosis** |  |  |  |  |  |  |  |
| 1 month-2 years | 14 (17.9%) | 46 (59.0%) | 18 (23.1%) | 0.113 | 13 (16.7%) | 65 (83.3%) | **0.043^*^** |
| 2-16 years | 30 (24.8%) | 53 (43.8%) | 38 (31.4%) |  | 9 (7.4%) | 112 (92.6%) |  |
| **Sex** |  |  |  |  |  |  |  |
| Male | 34 (28.1%) | 48 (39.7%) | 39 (32.2%) | **0.001^**^** | 15 (12.4%) | 106 (87.6%) | 0.452 |
| Female | 10 (12.8%) | 51 (65.4%) | 17 (21.8%) |  | 7 (9.0%) | 71 (91.0%) |  |
| **Seizure type** |  |  |  |  |  |  |  |
| Generalized onset | 30 (24.0%) | 61 (48.8%) | 34 (27.2%) | 0.507 | 18 (14.4%) | 107 (85.6%) | 0.059 |
| Focal onset | 10 (16.7%) | 31 (51.7%) | 19 (31.7%) |  | 3 (5.0%) | 57 (95.0%) |  |
| **ASMs therapy** |  |  |  |  |  |  |  |
| VPA monotherapy | 27 (25.5%) | 55 (51.9%) | 24 (22.6%) | 0.149 | 11 (10.4%) | 95 (89.6%) | 0.745 |
| Polytherapy | 17 (18.3%) | 44 (47.3%) | 32 (34.4%) |  | 11 (11.8%) | 82 (88.2%) |  |

Abbreviations:

ASMs = antiseizure medications;

VPA = valproic acid

* *p* < 0.05, ** *p* < 0.01

**Table S2** Distribution of *TPH2* rs1386494 genotypes in uncontrolled seizure and seizure-free groups

| **Genotype** | **Uncontrolled Seizure**  **(n = 77)** | **Seizure-Free**  **(n = 122)** | **OR (95%CI)** | ***p*-Value** |
| --- | --- | --- | --- | --- |
| TC vs. CC | 8 (10.4%) | 14 (11.5%) | 1.00 | 0.81 |
|  | 69 (89.6%) | 108 (88.5%) | 0.89 (0.36-2.24) |  |

**Table S3** Distribution of *TPH2* rs1386494 genotypes in the groups of patients with and without nADRs

| **Genotype** | **Patients with nADRs (n = 61)** | **Patients without nADRs (n = 147)** | **OR (95%CI)** | ***p*-Value** |
| --- | --- | --- | --- | --- |
| TC vs. CC | 10 (16.4%) | 14 (9.5%) | 1.00 | 0.17 |
|  | 51 (83.6%) | 133 (90.5%) | 1.86 (0.78-4.46) |  |

Abbreviations:

nADRs = neurological adverse drug reactions

**Table S4** Distribution of *TPH2* rs1386494 genotypes in the groups of patients with and without gADRs

| **Genotype** | **Patients with gADRs (n = 69)** | **Patients without gADRs (n = 139)** | **OR (95%CI)** | ***p*-Value** |
| --- | --- | --- | --- | --- |
| TC vs. CC | 7 (10.1%) | 17 (12.2%) | 1.00 | 0.65 |
|  | 62 (89.9%) | 122 (87.8%) | 0.81 (0.32-2.06) |  |

Abbreviations:

gADRs = gastrointestinal adverse drug reactions

**Table S5** Distribution of *TPH2* rs1386494 genotypes in the groups of patients with and without weight gain

| **Genotype** | **Patients with weight gain (n = 58)** | **Patients without weight gain (n = 150)** | **OR (95%CI)** | ***p*-Value** |
| --- | --- | --- | --- | --- |
| TC vs. CC | 9 (15.5%) | 15 (10.0%) | 1.00 | 0.28 |
|  | 49 (84.5%) | 135 (90.0%) | 1.65 (0.68-4.02) |  |

**Table S6** Distribution of *TPH2* rs4570625 genotypes in the groups of patients with and without gADRs

| **Genetic Model** | **Genotype** | **Patients with gADRs (n = 69)** | **Patients without gADRs (n = 139)** | **OR (95%CI)** | ***p*-Value** |
| --- | --- | --- | --- | --- | --- |
| Allele contrast | G vs. T | 68 (49.3%) | 127 (45.7%) | 1.00 | 0.49 |
|  |  | 70 (50.7%) | 151 (54.3%) | 1.15 (0.77-1.74) |  |
| Codominant | GG vs. GT vs. TT | 14 (20.3%) | 32 (23.0%) | 1.00 | 0.19 |
|  |  | 40 (58.0%) | 63 (45.3%) | 0.69 (0.33-1.45) |  |
|  |  | 15 (21.7%) | 44 (31.7%) | 1.28 (0.54-3.03) |  |
| Dominant | GG vs. GT+TT | 14 (20.3%) | 32 (23.0%) | 1.00 | 0.65 |
|  |  | 55 (79.7%) | 107 (77.0%) | 0.85 (0.42-1.73) |  |
| Recessive | GG+GT vs. TT | 54 (78.3%) | 95 (68.3%) | 1.00 | 0.13 |
|  |  | 15 (21.7%) | 44 (31.7%) | 1.67 (0.85-3.27) |  |
| Overdominant | GG+TT vs. GT | 29 (42.0%) | 76 (54.7%) | 1.00 | 0.09 |
|  |  | 40 (58.0%) | 63 (45.3%) | 0.60 (0.34-1.08) |  |

Abbreviations:

gADRs = gastrointestinal adverse drug reactions

**Table S7** Distribution of *TPH2* rs4570625 genotypes in the groups of patients with and without weight gain

| **Genetic Model** | **Genotype** | **Patients with weight gain**  **(n = 58)** | **Patients without weight gain**  **(n = 150)** | **OR (95%CI)** | ***p*-Value** |
| --- | --- | --- | --- | --- | --- |
| Allele contrast | G vs. T | 61 (52.6%) | 134 (44.7%) | 1.00 | 0.15 |
|  |  | 55 (47.4%) | 166 (55.3%) | 1.37 (0.89-2.11) |  |
| Codominant | GG vs. GT vs. TT | 16 (27.6%) | 30 (20.0%) | 1.00 | 0.35 |
|  |  | 29 (50.0%) | 74 (49.3%) | 1.36 (0.65-2.86) |  |
|  |  | 13 (22.4%) | 46 (30.7%) | 1.89 (0.80-4.48) |  |
| Dominant | GG vs. GT+TT | 16 (27.6%) | 30 (20.0%) | 1.00 | 0.24 |
|  |  | 42 (72.4%) | 120 (80.0%) | 1.52 (0.76-3.07) |  |
| Recessive | GG+GT vs. TT | 45 (77.6%) | 104 (69.3%) | 1.00 | 0.23 |
|  |  | 13 (22.4%) | 46 (30.7%) | 1.53 (0.75-3.11) |  |
| Overdominant | GG+TT vs. GT | 29 (50.0%) | 76 (50.7%) | 1.00 | 0.93 |
|  |  | 29 (50.0%) | 74 (49.3%) | 0.97 (0.53-1.79) |  |
